# Foot type symmetry and change of foot structures from sitting to standing conditions

**DOI:** 10.1186/1757-1146-7-S1-A34

**Published:** 2014-04-08

**Authors:** Howard Hillstrom, Jinsup Song, Michael Neary, William Brechue, Rebecca A Zifchock, Steven Svoboda, Marian T Hannan

**Affiliations:** 1 Hospital for Special Surgery, New York, New York, USA; 2 Temple University School of Podiatric Medicine, Philadelphia, Pennsylvania, USA; 3 United States Military Academy, West Point, New York, USA; 4 Hebrew Senior Life, Harvard Medical School, Boston, USA

## Introduction

Foot symmetry and change in foot structure as a function of weight bearing status have not been investigated in a large cohort study. The foot structure of 1,054 incoming cadets at the US Military Academy (172 female, 18.5±1.1 years, 24.5±3.0 kg/m^2^) was examined. Arch Height Index (AHI) was assessed in sitting and standing condition, and its value was used to classify each foot into 3 foot types as previously described [1].

## Method

Based on standing AHI, 68.1%, 24.5%, and 7.5% of the study subjects’ left foot was categorized into planus, neutral, and cavus foot types, respectively. An asymmetrical foot type was observed in 28.6% of subjects in sitting and 23.6% standing conditions. Foot length increased from sitting to standing conditions; this change was significantly greater in cavus and neutral foot type groups than the planus group. In contrast, arch height flexibility (AHF) was significantly greater in the planus group than both cavus and neutral foot type groups.

## Results

Results of this study suggest the importance of controlling for weight bearing status when assessing foot structure or fitting footwear. Given that about a quarter of participants demonstrated an asymmetrical foot type, findings also suggest the importance of assessing both feet independently. Table 1.

**Table 1:** Mean arch height flexibility and change in foot length across the 3 foot type groups

	Cavus	Neutral	Planus	P-value
N (female)	53 (5)	184 (34)	711 (133)	

AHF (mm/kN)	13.2 ± 7.4	14.8 ± 7.4	16.6 ± 7.4	0.0001 ^a,c^

ΔFoot Length (mm)	4.8 ± 2.6	4.3 ± 2.2	3.6 ± 2.1	<.0001 ^a,c^

Arch height flexibility = [(arch height in sitting – arch height in standing) / (0.4 * body weight)]. A significant difference (P<0.05) was observed between ^a^ cavus and planus foot types and ^c^ between neutral and planus foot types.
